# Real-World Use of Cefiderocol for Multidrug-Resistant Gram-Negative Infections in Critically Ill ICU Patients: A Single-Center Case Series

**DOI:** 10.3390/pathogens15080786

**Published:** 2026-07-24

**Authors:** Stelian Adrian Ritiu, Adelina Baloi, Marius Papurica, Dorel Sandesc, Daiana Toma, Claudiu Rafael Barsac, Sonia Elena Popovici, Madalina Butas, Ana-Maria-Ionela Botoaca, Ovidiu Bedreag

**Affiliations:** 1 Faculty of Medicine, Victor Babes University of Medicine and Pharmacy, 300041 Timisoara, Romaniabedreag.ovidiu@umft.ro (O.B.); 2 Clinic of Anaesthesia and Intensive Care, Emergency County Hospital Pius Brinzeu, 300723 Timisoara, Romania; 3 Doctoral School, Victor Babes University of Medicine and Pharmacy Timisoara, Eftimie Murgu Square 2, 300041 Timisoara, Romania; popovici.sonia@umft.ro (S.E.P.);; 4 Anaesthesia and Intensive Care Research Center (CCATITM), Victor Babes University of Medicine and Pharmacy, 300041 Timisoara, Romania

**Keywords:** microbiological eradication, cefiderocol, ICU, limited option antibiotic therapy, critically ill patients, carbapenem resistance, polymicrobial infection

## Abstract

Background/Objectives: Carbapenem-resistant Gram-negative (CR-GN) infections are associated with high mortality in intensive care units (ICUs), with limited therapeutic options. We aimed to evaluate microbiological and clinical outcomes associated with cefiderocol in critically ill patients with multidrug-resistant (MDR) Gram-negative infections. Methods: This retrospective, single-center case series included 25 critically ill patients with carbapenem-resistant or multidrug-resistant Gram-negative infections treated with cefiderocol between May 2024 and October 2025 in a mixed ICU of a tertiary hospital in Romania. Microbiological cure was defined as eradication of the designated target pathogen at the end of therapy. Clinical evolution was assessed using the Acute Physiology and Chronic Health Evaluation II (APACHE II) and Sequential Organ Failure Assessment (SOFA) scores, as well as inflammatory biomarkers including white blood cell count (WBC), C-reactive protein (CRP), and procalcitonin (PCT). Results: *Klebsiella pneumoniae* was the predominant pathogen, identified in 23 of 25 patients (92%), followed by *Acinetobacter baumannii* (11/25, 44%) and *Pseudomonas aeruginosa* (6/25, 24%). As multiple pathogens were co-isolated in 16 patients (64%), percentages exceed 100% and are reported as proportions of patients rather than of total isolates. Microbiological cure was achieved in 68% of patients. Mortality was markedly higher in patients without microbiological eradication (88% vs. 35%). Higher baseline APACHE II (HR 1.18, 95% CI 1.02–1.37) and SOFA scores (HR 1.33, 95% CI 1.07–1.65) were associated with increased mortality. Early initiation of cefiderocol (≤10 days from ICU admission) was associated with improved microbiological and clinical outcomes compared to delayed treatment. Reductions in severity scores and inflammatory markers, including C-reactive protein (CRP) and procalcitonin (PCT), were observed during therapy. Pathogen type and combination therapy were not clearly associated with outcomes in this cohort. Conclusions: Cefiderocol was observed in association with clinically relevant rates of microbiological eradication and improvements in clinical parameters in critically ill patients with MDR Gram-negative infections. Outcomes appeared to be primarily determined by baseline disease severity, while earlier initiation of therapy was associated with more favourable microbiological and clinical outcomes, although causality cannot be established given the observational design and absence of a comparator group. These findings are hypothesis-generating and supportive of a potential role for cefiderocol as a salvage option in high-risk ICU populations, pending validation in larger, prospective, controlled studies.

## 1. Introduction

Antimicrobial resistance (AMR) is a growing global health threat, with multidrug-resistant (MDR) Gram-negative (GN) infections causing more than one million deaths worldwide in 2019 [1].

European AMR surveillance data for 2021 indicated substantially higher resistance rates to third-generation cephalosporins and carbapenems in *Klebsiella pneumoniae* compared with *Escherichia coli*, with carbapenem resistance (CR) also frequently reported in *Pseudomonas aeruginosa* and *Acinetobacter* spp., particularly in Southern and Eastern Europe [2]. Infections with CR GN bacteria are linked to poor clinical outcomes and elevated mortality rates, largely due to limited therapeutic options and the complexity of management in critically ill patients in intensive care settings [3].

While CR *Klebsiella pneumoniae* (CR-Kp) remains a major hospital threat because of carbapenemase production, other non-fermenting GN pathogens, particularly *Pseudomonas* spp. and *Acinetobacter* spp. are increasingly implicated in difficult-to-treat and persistent infections [4]. These infections are frequently associated with recurrence, prolonged hospitalization, increased healthcare costs, and a substantially higher mortality risk [5]. Recently, the World Health Organization published the Bacterial Pathogen Priority List, classifying carbapenem-resistant Enterobacterales (CR-E) and *Acinetobacter baumannii* (CR-AB) as critical priority pathogens and *Pseudomonas aeruginosa* (CR-PA) as a high-priority pathogen. This classification aims to guide research, the development of new antimicrobial agents, and public health strategies against antimicrobial resistance, based on factors such as resistance, virulence, transmissibility, and limited treatment options [6].

Cefiderocol represents a structurally novel siderophore cephalosporin that exploits the bacterial iron acquisition system as a Trojan horse mechanism: its catechol moiety chelates ferric iron and facilitates active transport into the bacterial cell via siderophore uptake pathways, thereby bypassing the outer membrane permeability barrier that limits the entry of conventional beta-lactams in carbapenem-resistant organisms [7]. Once intracellular, cefiderocol inhibits penicillin-binding proteins and demonstrates stability against serine beta-lactamases, metallo-beta-lactamases (MBLs), and extended-spectrum beta-lactamases (ESBLs), including the most clinically relevant carbapenemases such as NDM, OXA-48, and VIM [8,9,10,11,12]. This dual mechanism—active cellular penetration combined with broad beta-lactamase stability—distinguishes cefiderocol from existing beta-lactam/beta-lactamase inhibitor combinations and underpins its activity against pathogens with complex, co-existing resistance mechanisms, as frequently encountered in ICU settings. The European Medicines Agency approved cefiderocol in April 2020 for infections caused by Gram-negative bacteria (GNB) with limited treatment options. Clinical trials, complemented by real-world studies, have demonstrated its efficacy in complicated urinary tract infections, nosocomial pneumonia, bloodstream infections, and other severe GN infections caused by resistant pathogens [13,14]. Its use is recommended by current European guidelines, particularly when susceptibility testing is positive and only limited alternative treatment options are available [15,16].

However, several important concerns and unresolved questions surrounding cefiderocol warrant consideration. The CREDIBLE-CR trial, despite demonstrating microbiological activity, reported numerically higher all-cause mortality in the cefiderocol arm compared with best available therapy (49% vs. 34%), raising concerns about clinical efficacy in the most severely ill patients, particularly those infected with Acinetobacter baumannii [13]. Furthermore, the emergence of resistance to cefiderocol during therapy has been documented in real-world settings, particularly in patients with prolonged courses or suboptimal pharmacokinetic/pharmacodynamic target attainment, underscoring the importance of appropriate dosing and susceptibility monitoring [17,18]. The role of combination therapy with cefiderocol remains controversial; while in vitro synergy data are promising, clinical evidence supporting the superiority of combination over monotherapy regimens is currently insufficient. Finally, existing real-world evidence is largely derived from small, retrospective, single-center case series with heterogeneous patient populations, limiting the generalizability of findings and the ability to draw definitive conclusions regarding efficacy and safety [19]. These considerations highlight the need for prospective, controlled studies to better define the optimal use of cefiderocol in critically ill patients.

In Romania, AMR is a major concern in hospital settings [20]. Hospital-based surveillance and epidemiological studies indicate that *Klebsiella* spp., *Acinetobacter* spp., and *E. coli* are among the most frequently isolated GN pathogens in intensive care units (ICUs), with a significant proportion of strains exhibiting MDR and carbapenem resistance [21]. In critically ill patients, disease severity is commonly assessed using validated scoring systems. The Acute Physiology and Chronic Health Evaluation II (APACHE II) score quantifies severity of illness based on physiological parameters, age, and chronic health status recorded within the first 24 h of ICU admission, with higher scores correlating with increased risk of in-hospital mortality. The Sequential Organ Failure Assessment (SOFA) score evaluates the degree of organ dysfunction across six organ systems—respiratory, coagulation, hepatic, cardiovascular, renal, and neurological—and is widely used both as a prognostic tool and as a surrogate marker of clinical trajectory in ICU patients. Both scores were used in this study to characterize baseline disease severity and to monitor clinical evolution during cefiderocol therapy. However, several key knowledge gaps remain. First, real-world data on cefiderocol use in Eastern European ICU settings are scarce, limiting the applicability of findings from Western European or North American cohorts to high-resistance epidemiological contexts such as Romania. Second, existing real-world studies have rarely examined the simultaneous impact of baseline disease severity, timing of therapy initiation, and polymicrobial infection patterns on both microbiological and clinical outcomes. Third, predictors of treatment response in critically ill patients with polymicrobial MDR infections receiving cefiderocol as salvage therapy remain poorly characterized.

The present study aims to address these gaps by reporting the real-world experience of a Romanian tertiary ICU with cefiderocol in a consecutive cohort of critically ill patients with MDR Gram-negative infections, with a focus on microbiological eradication rates, clinical trajectory, and predictors of outcome.

## 2. Results

### 2.1. Patient Characteristics at Baseline

A total of 25 critically ill patients (14 male) were included in the analysis. The mean time from hospital admission to microbiological confirmation of infection (positive culture with antimicrobial susceptibility results) was 38 days (median 21 days, range 6 to 342). Cefiderocol therapy was initiated on the day of infection confirmation in 8 patients, on the following day in 13 patients and 2 days after confirmation in 4 patients (targeted antibiotic treatment based on antibiogram result The majority of patients received combination antibiotherapy (16/25, 64%), most frequently with colistin (32%) and linezolid (20%). Linezolid was administered specifically to target co-isolated Gram-positive organisms (e.g., *Enterococcus* spp.) rather than the primary Gram-negative pathogens. Most patients (72%) required vasopressor support. All patients had received prior antibiotic therapy before cefiderocol initiation, most commonly meropenem (68%), amikacin (52%), colistin (52%), and linezolid (40%). No patients received extracorporeal support (ECMO or continuous renal replacement therapy) during cefiderocol treatment, and no episodes of acute kidney injury (AKIN criteria) were documented during therapy. No patients had documented immunosuppressive conditions or therapy (e.g., transplant, active chemotherapy, chronic corticosteroid use, or neutropenia) at baseline. Other clinical characteristics of this case series are detailed in Table 1.

### 2.2. Microbiological Profile and Resistance Patterns Before Cefiderocol Initiation

A total of 52 pathogen isolates were identified across the 25 patients. To address this, we performed a retrospective review of original clinical microbiology reports to reconstruct, at the isolate level, the specimen type, the designated target pathogen (defined in Section 4.3), and treatment outcome for each patient; this reconstruction is presented in Table 2. Unless otherwise specified, results throughout this manuscript are reported at the patient level (i.e., proportion of patients harbouring a given pathogen or resistance gene), given that 16 patients (64%) had polymicrobial infections; isolate-level counts are reported only in this introductory sentence.

Unless otherwise specified, results throughout this manuscript are reported at the patient level (i.e., proportion of patients harbouring a given pathogen or resistance gene), given that 16 patients (64%) had polymicrobial infections; isolate-level counts are reported only in this introductory sentence. All isolated pathogens were confirmed as multidrug-resistant (MDR) by antimicrobial susceptibility testing and multiplex PCR resistance gene profiling (Unyvero). Klebsiella pneumoniae was the predominant causative pathogen, identified in 23 of 25 patients (92%), followed by Acinetobacter baumannii (11/25, 44%) and Pseudomonas aeruginosa (6/25, 24%). Additional Gram-negative pathogens identified included Enterobacter cloacae (2 patients), Stenotrophomonas maltophilia (2 patients), Proteus spp., Escherichia coli, Klebsiella oxytoca, and Serratia marcescens (1 patient each). Among the co-isolated organisms, Enterococcus faecalis, Enterococcus faecium, Cutibacterium acnes, and Bacteroides fragilis were identified in individual patients within the context of polymicrobial infections. As cefiderocol has no clinically relevant activity against Gram-positive or anaerobic organisms, these pathogens were not considered target pathogens for the purposes of microbiological cure assessment and were managed with separate targeted agents according to susceptibility results. Cutibacterium acnes was considered a likely contaminant in the clinical context in which it was isolated, consistent with its recognized role as a common skin-flora contaminant in clinical cultures, and was not treated as a causative pathogen (see Section 4.2 for specimen types used for microbiological assessment). Since 16 patients (64%) had polymicrobial infections with two or more co-isolated pathogens, individual pathogen frequencies exceed 100% and are reported as proportions of patients rather than of total isolates. Screening cultures at admission in ICU were negative in 40% of patients. Of those positive at screening extended-spectrum beta-lactamase (ESBL)-producing organisms were detected in 40% of samples, carbapenem-resistant Enterobacterales (CRE) in 32%, methicillin-resistant *Staphylococcus aureus* (MRSA) in 20%, and vancomycin-resistant *Enterococcus* (VRE) in 16% of cases.

Resistance gene profiling by PCR testing of different pathogenic samples from infected patients revealed a high prevalence of carbapenemase and ESBL-related genes, NDM was detected in 52% of isolates/patients and OXA-48 in 48%, followed by CTX-M (40%) and TEM (28%) (Table 3).

#### Type of Infection

Respiratory infections were the most common in half of patients (52%), followed by abdominal infections, with other types reported less frequently (Table 1). The correlation between infection category, corresponding clinical specimen type, and predominant pathogens recovered is presented in (Table 2)**.** More than half of patients underwent at least one surgical intervention, with 56% requiring surgery during hospitalization, majority of them before ICU admission.

### 2.3. Microbiological and Clinical Cure with Cefiderocol Treatment

**Figure 1:**
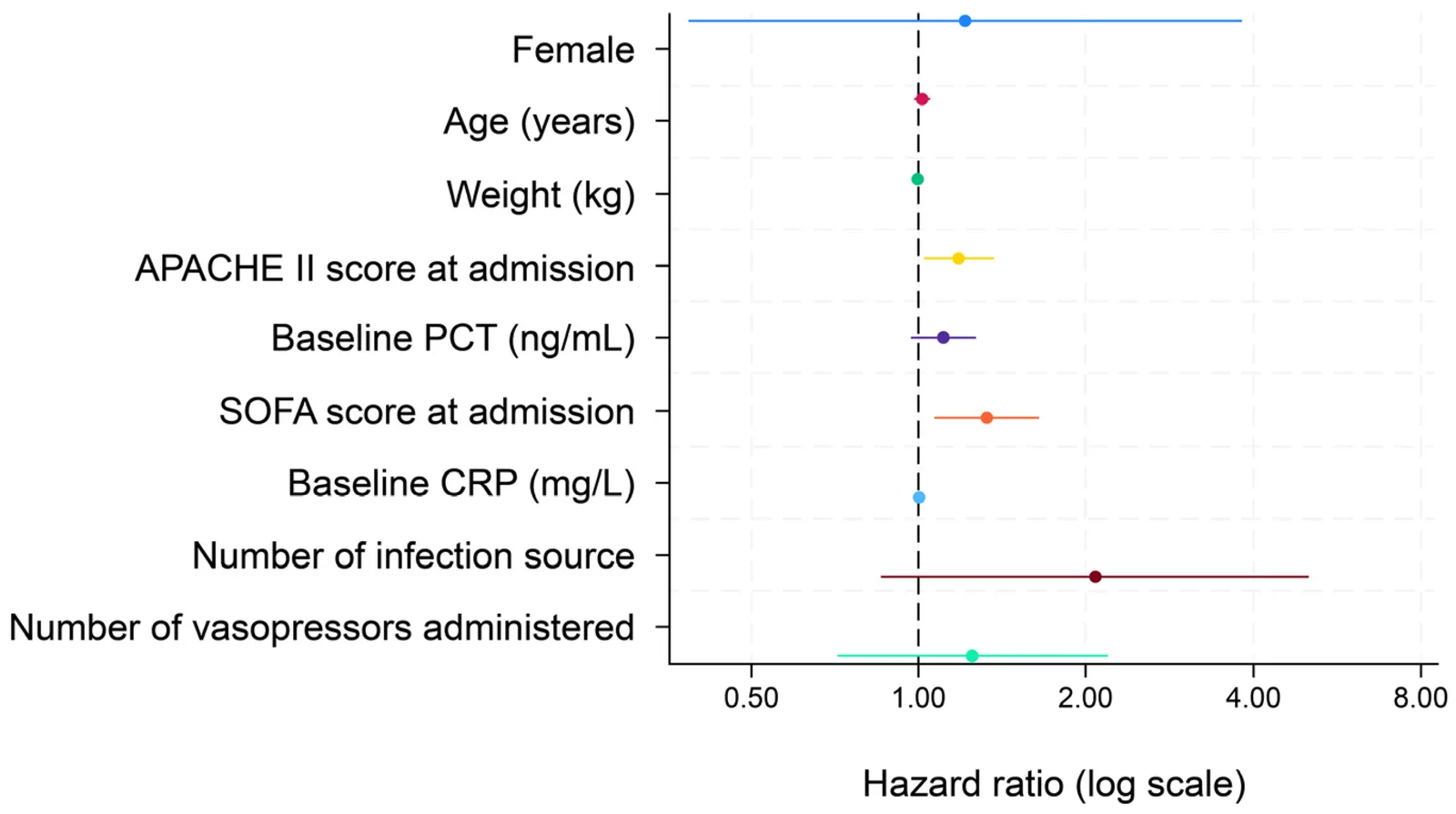
Forest plot of hazard ratios for variables associated with overall survival; points represent hazard ratios and horizontal lines indicate 95% confidence intervals; the dashed vertical line denotes no effect (hazard ratio = 1).

Given the limited sample size, the following exploratory subgroup and interaction analyses should be interpreted as hypothesis-generating; detailed results are presented in Supplementary Materials (Figures S1–S7), with only the principal survival analysis (Cox proportional hazards) retained in the main text (Figure 1). Microbiological effectiveness at the end of cefiderocol treatment was achieved in 17 of 25 patients (68%) (Table S1). Of them, 11 patients (65%) were discharged and 6 (35%) died during hospitalization. In contrast, in patients without microbiological response, only 1 of 8 (13%) survived and 7 of 8 (88%) died in hospital. It should be noted that patients without microbiological eradication were also more likely to have greater underlying disease severity and organ dysfunction; therefore, this association should not be interpreted as evidence of a direct causal effect of microbiological failure on mortality.

The timing of cefiderocol initiation relative to ICU admission appeared to influence both microbiological and clinical outcomes. Initiation within the first 10 days after ICU admission was associated with higher rates of microbiological cure (6/7) and survival (4/7) compared with later initiation (descriptive comparison; not formally tested given small subgroup sizes). In contrast, delayed initiation beyond 20 days after ICU admission was associated with lower rates of favourable outcomes, with variability observed in both microbiological response and survival across subgroups.

Exploratory analysis suggested a potential interaction between infection type and age, with divergent age-related trends in probability of microbiological cure across infection categories (respiratory and gastrointestinal/intra-abdominal). Given the small size of each infection subgroup, these interactions should be regarded as hypothesis-generating rather than robust predictors of outcome, and are presented in Supplementary Figure S1 without further mechanistic interpretation. Regarding screening culture results, the ESBL-positive screening cultures were significantly associated with reduced microbiological effectiveness (OR = 0.06, *p* < 0.05). An age-dependent effect was observed in patients with negative screening cultures (OR = 0.14, *p* < 0.05). CRE- and MRSA-positive screening cultures were not associated with a microbiological response (Figure S2).

Hemodynamic instability appeared to influence treatment efficiency. The number of vasopressors required during ICU stay was inversely associated with microbiological response. An increasing number of vasopressors was associated with lower treatment effectiveness (OR = 0.12, *p* < 0.05), reflecting the impact of greater hemodynamic instability and disease severity. The type of vasopressor did not influence effectiveness, with one exception, vasopressin, whose relative effectiveness became comparable to other agents only at age of 45 years and over.

A shorter time from admission to infection confirmation was associated with higher treatment success (OR = 0.86, *p* < 0.05), and longer overall hospitalization showed a trend toward increased probability of success (OR = 1.13, *p* < 0.10). The length of stay in the ICU was not significantly associated with the outcome.

Microbiological effectiveness was not associated with pathogen type, previous antibiotic therapy, or concomitant antibiotic use. In a descriptive comparison, microbiological cure rates were similar between patients receiving cefiderocol monotherapy (6/9, 67%) and combination therapy (11/16, 69%; Fisher exact *p* = 1.0). Survival was numerically higher among patients receiving combination therapy (10/16, 62.5%) compared with monotherapy (2/9, 22%; Fisher exact *p* = 0.097), although this difference should be interpreted cautiously given the small sample size and potential confounding by indication.

### 2.4. Evolution of Severity Scores and Inflammatory Markers

Patient severity decreased during ICU stay, as reflected by declining APACHE II scores and in mean SOFA score (from 4.3 ± 2.9 to 3.2 ± 3.9; mean ± SD; paired *t*-test, mean difference 1.04, 95% CI 0.05–2.03; *p* = 0.04). As no comparator group was available, this improvement cannot be attributed specifically to cefiderocol and may partly reflect the broader trajectory of critical illness and concurrent ICU management during the same interval.

Clinical effectiveness was evaluated based on changes in disease severity scores (APACHE II and SOFA) and inflammatory markers (white blood cells [WBC], C-reactive protein [CRP], and procalcitonin [PCT]) measured at the end of cefiderocol therapy. A reduction in disease severity was observed during cefiderocol administration (Table 4).

#### 2.4.1. Evolution of APACHE II Scores

Final APACHE II scores decreased during cefiderocol therapy, with higher baseline scores associated with increased mortality risk. Delayed confirmation of infection was associated with higher final APACHE II scores (*p* < 0.001), while longer overall hospitalization was associated with lower final scores (*p* < 0.01), likely reflecting progressive clinical stabilization in survivors. Baseline disease severity, rather than infection type or pathogen characteristics, emerged as the primary determinant of clinical trajectory. Exploratory analysis also suggested a gender-specific pattern in treatment response relative to baseline APACHE II, presented in Supplementary Figure S4. It should be noted that APACHE II was originally developed as a baseline prognostic score rather than a tool for longitudinal monitoring; serial APACHE II changes reported here should therefore be interpreted as a pragmatic surrogate of clinical trajectory rather than a validated repeated-measures instrument.

#### 2.4.2. Evolution of SOFA Scores

SOFA scores decreased significantly during cefiderocol therapy (from 4.3 ± 2.9 to 3.2 ± 3.9; mean difference 1.04, 95% CI 0.05–2.03; *p* = 0.04). Baseline SOFA was the primary predictor of final SOFA scores (*p* < 0.001), with delayed infection confirmation associated with higher final scores.

A significant interaction between baseline SOFA and PCT levels was observed (*p* < 0.05), suggesting that inflammatory burden at admission may modulate organ dysfunction trajectory; given the small subgroup size, this exploratory finding is presented in Supplementary Figure S5.

#### 2.4.3. Evolution of CRP

CRP levels decreased significantly during therapy, with a mean reduction of 80.1 mg/L from initiation to the end of antibiotic therapy (95% CI 32.9–127.4; *p* = 0.002), suggesting a favourable inflammatory response.

A significant interaction between baseline CRP and age was observed (*p* < 0.05), with higher baseline CRP values associated with higher predicted final CRP levels; given the exploratory nature of this interaction, results are presented in Supplementary Figure S6.

#### 2.4.4. Evolution of PCT

PCT levels decreased in patients who achieved microbiological cure and increased with delayed infection confirmation (*p* < 0.05). Higher baseline APACHE II (*p* < 0.01) and CRP values (*p* < 0.05) were associated with higher PCT levels at the end of cefiderocol therapy. A gender-specific difference in final PCT values was also observed (*p* < 0.05). WBC dynamics were primarily driven by baseline inflammatory status, without consistent independent predictors beyond baseline CRP (*p* < 0.10). Notably, median WBC increased slightly over the course of therapy despite parallel reductions in CRP and PCT; this dissociation may reflect the multifactorial drivers of leukocytosis in critically ill patients (e.g., stress response, corticosteroid use, or non-infectious inflammatory triggers) beyond the resolution of the primary infection. Exploratory modelling identified consistent gender-specific patterns in probability of microbiological cure relative to baseline disease severity and inflammatory markers across all parameters examined; these hypothesis-generating findings are presented in Supplementary Figure S4.

### 2.5. Recovery vs. Death

Exploratory analysis of recovery versus death identified several predictors of outcome. Lower baseline APACHE II scores were associated with a higher probability of cure, while SOFA scores, CRP, and PCT did not independently predict recovery. A significant age–weight interaction was observed, with younger patients with higher body weight showing a greater likelihood of cure; given the exploratory nature of this interaction, results are presented in Supplementary Figure S7. ESBL-positive screening status was associated with a markedly reduced probability of microbiological cure (OR = 0.03, *p* < 0.01), while surgical intervention was associated with increased cure probability (OR = 33.25, *p* < 0.01).

In univariable Cox proportional hazards analysis, higher baseline APACHE II (HR = 1.18, 95% CI: 1.02–1.37, *p* < 0.05) and SOFA scores (HR = 1.33, 95% CI: 1.07–1.65, *p* < 0.05) were associated with increased mortality risk (Figure 1). Age, gender, weight, inflammatory markers, and vasopressor use did not independently influence overall survival. The proportional hazards assumption was confirmed using Schoenfeld residuals. Given the limited sample size, all findings should be considered hypothesis-generating.

## 3. Discussion

MDR Gram-negative infections represent a major challenge in the ICU setting due to limited therapeutic options and high mortality rates. In this real-world case series, cefiderocol was used as a last-resort treatment option in critically ill patients with predominantly carbapenem-resistant Gram-negative infections. Our findings suggest clinically relevant rates of microbiological eradication and improvement in severity scores, with clinical outcomes largely determined by baseline disease severity rather than pathogen characteristics.

The resistance gene profile of our cohort is particularly noteworthy in the context of cefiderocol’s mechanism of action. NDM was detected in 52% of patients and OXA-48 in 48%, frequently co-occurring within the same isolate—a pattern consistent with the high-resistance epidemiology of Eastern European ICUs and representing one of the most therapeutically challenging combinations encountered in clinical practice. NDM-type metallo-beta-lactamases hydrolyze virtually all beta-lactam classes, including carbapenems, and are not inhibited by commercially available beta-lactamase inhibitors, leaving few systemic treatment options. OXA-48-type carbapenemases confer carbapenem resistance through a distinct serine-based mechanism and are increasingly co-produced with ESBLs such as CTX-M, detected in 40% of our patients. Cefiderocol’s stability against both MBL and serine carbapenemase hydrolysis, combined with its iron transporter-mediated cellular entry that circumvents outer membrane permeability loss, provides a mechanistic basis for the microbiological activity observed in this highly resistant cohort. The 68% microbiological cure rate achieved despite the predominance of NDM- and OXA-48-producing isolates supports the clinical relevance of this mechanism in real-world conditions, where co-resistance to all available alternatives is common. This is particularly relevant given that NDM-producing isolates are typically resistant to all currently available beta-lactam/beta-lactamase inhibitor combinations, including ceftazidime–avibactam and meropenem–vaborbactam, leaving polymyxins, tigecycline, or eravacycline as the main alternatives—agents associated with either inferior efficacy or greater toxicity in critically ill patients. Cefiderocol therefore addresses a specific therapeutic gap for metallo-beta-lactamase-producing organisms in this population.

Although pivotal trials such as CREDIBLE-CR, APEKS-NP and APEKS-cUTI have established the efficacy of cefiderocol in selected patient populations, evaluating its performance in routine clinical practice remains essential [12,13,22]. Microbiological cure was achieved in 68% of patients, consistent with real-world studies reporting rates of 64–80%. Pathogen type was not associated with microbiological eradication, supporting the broad-spectrum activity of cefiderocol. The predominant pathogens in our cohort—Klebsiella pneumoniae (92%), Acinetobacter baumannii (44%), and Pseudomonas aeruginosa (24%)—exhibited MDR profiles, including XDR and PDR phenotypes, reflecting the high resistance burden in our setting [4]. Earlier initiation of cefiderocol (≤10 days from ICU admission) was associated with more favourable outcomes, while delayed initiation (>20 days) was linked to less consistent responses.

The APACHE II and SOFA scores were used pragmatically as surrogate markers of clinical trajectory, acknowledging that they were not originally designed for longitudinal monitoring of treatment response. Both scores decreased during cefiderocol therapy, accompanied by a marked reduction in CRP levels, suggesting a favourable systemic response. In contrast, WBC and PCT showed more heterogeneous dynamics, likely reflecting baseline inflammatory burden and individual host resilience. Baseline disease severity was the primary determinant of clinical outcomes, with higher admission scores associated with increased mortality risk and lower probability of clinical improvement.

Temporal factors were associated with clinical course. Delayed confirmation of infection correlated with higher final severity scores and inflammatory markers, underscoring the importance of early diagnosis and timely targeted therapy. Conversely, longer overall hospital stay was linked to lower final APACHE II and CRP values, possibly reflecting progressive stabilization among patients who survived the initial critical phase. ICU length of stay was not related to microbiological or inflammatory outcomes.

The strongest link between microbiological and clinical outcomes in our cohort was the marked mortality difference according to eradication status (88% in patients without microbiological cure vs. 35% in those who achieved cure), reinforcing microbiological eradication as the most clinically relevant surrogate outcome in this population. We observed that mortality occurred predominantly among patients who failed to achieve microbiological eradication. This finding supports the association between persistent infection and adverse outcomes and underscores the critical role of effective infection control in this patient population. Notably, 6 of 17 patients who achieved microbiological eradication also died during hospitalization. All deaths in patients without microbiological eradication were infection-related, whereas deaths among patients with microbiological eradication were attributed to non-infectious causes. These observations suggest that, in critically ill populations, outcomes are often driven by host factors and multi-organ failure rather than microbiological response alone. These findings suggest that eradication of the pathogen does not necessarily translate into survival in critically ill patients.

Combination antimicrobial therapy was frequently used in this cohort, particularly in patients with more severe infections, consistent with real-world ICU practice. Colistin and linezolid were the most commonly co-administered agents. International guidelines (ESCMID and IDSA) recommend combination regimens in selected high-risk carbapenem-resistant infections, particularly those caused by *A. baumannii* [16,23]. Nevertheless, despite in vitro data suggesting potential synergistic effects of cefiderocol-based combinations, concomitant antibiotic therapy was not associated with improved microbiological response or survival, likely reflecting confounding by indication in more severely ill patients. These observations contribute to the ongoing debate regarding the clinical value of combination strategies in the era of novel β-lactams and siderophore cephalosporins [24,25,26,27]. Notably, survival was numerically higher among patients receiving combination therapy in our cohort, which may reflect confounding by indication—for example, hemodynamically unstable patients may have been managed with monotherapy—rather than a protective effect of combination regimens; this exploratory observation warrants cautious interpretation and further investigation in larger cohorts.

In our cohort, no treatment discontinuations or adverse events attributable to cefiderocol were observed. Prolonged hospitalization and death were observed in patients with more severe clinical status who failed to achieve microbiological eradication, suggesting that mortality was more likely related to infection severity rather than to cefiderocol treatment. These observations are consistent with prior clinical trials and real-world studies reporting a favourable safety profile for cefiderocol, with serious adverse events rarely requiring discontinuation [15,28,29,30].

### 3.1. Clinical Implications

These findings suggest that cefiderocol may be particularly relevant in critically ill patients with MDR Gram-negative infections when conventional therapeutic options are exhausted. Baseline disease severity, rather than pathogen type, was the primary determinant of outcome, emphasizing the importance of early initiation of targeted antimicrobial therapy before advanced organ dysfunction develops. Microbiological eradication was associated with improved survival, though it did not universally prevent death in this critically ill population, highlighting the role of host factors and multi-organ failure in determining final outcomes. The probability of treatment success appeared to decline beyond specific severity thresholds, reinforcing the need for timely intervention [31,32,33,34].

### 3.2. Limitations

Despite these encouraging observations, this study has several limitations. Its retrospective, single-center design limits the generalizability of the findings and may introduce selection bias. In particular, the high local prevalence of NDM- and OXA-48-producing organisms may limit direct extrapolation of these findings to settings with different resistance epidemiology, where the relative contribution of baseline severity versus pathogen-specific factors to outcomes may differ. The lack of a comparator arm precludes assessment of the comparative effectiveness of cefiderocol, and the small sample size reduces statistical power. Multiple regression models were explored with a limited number of events relative to the number of variables included; therefore, some associations should be interpreted as hypothesis-generating. Therapeutic drug monitoring of cefiderocol was not performed, as this is not part of routine clinical practice locally; this represents a limitation given the relevance of dose optimization in critically ill patients with variable renal clearance. Furthermore, in the absence of a comparator group, the clinical and microbiological improvements observed during cefiderocol therapy cannot be causally attributed to the drug itself. These findings may also reflect survivor bias (as more severely ill patients were more likely to die before completing therapy), the effect of concomitant antimicrobial and supportive therapies administered during the same interval, or the natural evolution of critical illness independent of antimicrobial treatment. Clinical evolution was assessed through continuous changes in severity scores and inflammatory markers rather than a standardized, predefined clinical response criterion, which limits comparability with studies using dichotomous response definitions.

Nevertheless, our findings provide real-world evidence on cefiderocol use in critically ill patients with limited therapeutic options. The detailed evaluation of severity scores and inflammatory marker dynamics during cefiderocol therapy enabled the exploration of potential predictive models in a highly complex ICU population. These observations may serve as a basis for future multicenter studies with larger sample sizes to validate our preliminary findings.

The epidemiological context of this study warrants specific consideration. Romania reports among the highest rates of carbapenem resistance in Europe, with NDM- and OXA-48-producing Enterobacterales, carbapenem-resistant Acinetobacter baumannii, and MDR Pseudomonas aeruginosa widely disseminated in hospital settings [4,5]. Recent in vitro surveillance data confirm that cefiderocol retains activity against a substantial proportion of clinical MDR Gram-negative isolates circulating in Romanian hospitals, providing microbiological support for its use as a salvage option in this setting [20,34]. These findings reinforce the importance of incorporating cefiderocol within structured antimicrobial stewardship frameworks—restricted to confirmed MDR infections with limited alternatives, supported by susceptibility testing, and accompanied by ongoing microbiological surveillance to detect early resistance emergence. Real-world evidence from high-resistance epidemiological contexts such as Romania is essential to complement data from Western European and North American cohorts, where resistance patterns and patient populations may differ substantially.

## 4. Materials and Methods

### 4.1. Design, Setting and Ethics Approval

This retrospective, single-center observational case series included 25 critically ill patients with severe MDR Gram-negative infections admitted to the ICU of the Emergency County Hospital “Pius Brînzeu”, Timișoara, between 1 May 2024 and 1 October 2025. The study was conducted in accordance with the Declaration of Helsinki and approved by the Institutional Review Board of the “Pius Brînzeu” Emergency County Clinical Hospital Timișoara (approval number 569/08.10.2025) and by the Institutional Ethics Committee for Scientific Research of the “Victor Babeș” University of Medicine and Pharmacy Timișoara (approval number 86/2025). The requirement for informed consent was waived due to the retrospective nature of the analysis.

Cefiderocol use at our institution is governed by an antimicrobial stewardship protocol requiring approval by the attending intensivist and infectious disease consultant. Prescription is restricted to documented MDR or carbapenem-resistant Gram-negative infections with confirmed or highly probable susceptibility to cefiderocol based on antimicrobial susceptibility testing and/or resistance gene profiling. All cases were reviewed at a weekly multidisciplinary antimicrobial stewardship meeting.

Demographic characteristics (age, gender, weight, and height), as well as type and site of infection, were collected at the start of cefiderocol treatment. Clinical parameters (APACHE II and SOFA scores, CRP and PCT levels) were collected during treatment. Microbiological cure was assessed at the end of cefiderocol therapy.

### 4.2. Treatment and Microbiology

Cefiderocol was administered intravenously at 2 g every 8 h as a 3 h infusion, with dose adjustments according to renal function (creatinine clearance), in line with the Summary of Product Characteristics. All patients received cefiderocol as the best available salvage therapy, either as monotherapy or in combination therapy, based on culture results and/or PCR detection of resistance genes. Screening cultures were performed on ICU admission. Microbiological cure was assessed by comparing pre-treatment and post-treatment cultures obtained from the relevant clinical specimens, including bronchoalveolar lavage or endotracheal aspirates for respiratory infections, blood cultures for bacteraemia, urine cultures for urinary tract infections, and wound/tissue swabs for skin and soft tissue infections. Follow-up cultures were collected at the end of the cefiderocol course. Pathogen identification was performed using MALDI-TOF MS (VITEK MS, bioMérieux, Marcy-l’Étoile, France), and antimicrobial susceptibility testing was performed using the disk diffusion method (Oxoid, Thermo Fisher Scientific, Basingstoke, UK) in all patients, with results interpreted according to EUCAST breakpoints. Resistance gene profiling was additionally conducted using a multiplex PCR assay (Unyvero, Curetis) on clinical samples obtained prior to cefiderocol initiation.

### 4.3. Definitions

MDR was defined as acquired non-susceptibility to at least one agent in three or more antimicrobial categories; XDR as non-susceptibility to at least one agent in all but two or fewer antimicrobial categories; and PDR as non-susceptibility to all agents in all antimicrobial categories [7].

Microbiological cure was defined as sterility (no growth) of a follow-up culture obtained from the same specimen type as the pre-treatment culture documenting the causative infection, assessed at the end of cefiderocol therapy. For the 20 patients in whom full isolate-level reconstruction was possible (Supplementary Table S1), this corresponded specifically to eradication of the designated target pathogen—the organism confirmed as carbapenem-resistant and cefiderocol-susceptible on antibiogram, for which cefiderocol was specifically initiated. For the remaining 5 patients (Patients 4, 9, 11, 14, and 18), for whom the original specimen-level microbiology report could not be retrieved, cure was assessed as sterility of the follow-up culture from the same specimen type as the treated infection, without isolate-level confirmation of the specific organism eradicated. In polymicrobial infections, co-isolated organisms (including Gram-positive species) were managed with concomitant targeted agents and were not included in this assessment. Clinical evolution was assessed as the change in severity scores (APACHE II, SOFA) and inflammatory markers (WBC, CRP, PCT) from cefiderocol initiation to the end of therapy, analyzed as continuous variables rather than as a predefined dichotomous outcome. No standardized categorical clinical response criteria were applied.

### 4.4. Statistical Analysis

Descriptive statistics were used to summarize the study population. Continuous variables were reported as mean ± standard deviation (SD) for approximately normally distributed data or as median and interquartile range (IQR) for non-normally distributed data. Categorical variables were expressed as counts and percentages. Given the limited sample size and the exploratory nature of this case series, inferential analyses were interpreted cautiously and considered hypothesis-generating. Model complexity was intentionally restricted to reduce the risk of overfitting. The choice of regression model was based on the type of dependent variable, including logistic regression for binary outcomes, linear regression for continuous outcomes, and Cox proportional hazards regression for time-to-event analyses. Tobit regression was used where appropriate for censored continuous outcomes.

First, exploratory logistic regression models were fitted using gender (male vs. female), age (years), and body weight (kg) as predictors. Subsequently, interaction terms between pairs of predictors were introduced. All models showed pseudo-R^2^ values ranging from 0.20 to 0.33 and area under curve (AUC) values ranging from 0.78 to 0.88. The interaction between age and gender was the only interaction term consistently associated with the outcome across models. As body weight did not demonstrate a meaningful contribution, it was excluded from subsequent analyses. Given the limited sample size, subsequent exploratory models retained age, gender, and their interactions core control variables (CCV). Each additional predictor was then entered separately into the model together with the CCV, including APACHE II and SOFA scores at hospitalization, CRP and PCT at initiation of antibiotic therapy, SOFA score at cefiderocol discontinuation, number and type of infection sources, hospitalization diagnoses, infection diagnoses, presence of *Acinetobacter baumannii* infection (the only positive culture with sufficient variation), history of surgical intervention, number of vasopressors administered, previous antibiotic therapy, time from hospitalization to treatment initiation, duration of hospitalization and length of intensive care unit stay. For each predictor, three exploratory models were assessed: as a main effect only, in interaction with age, and in interaction with gender. This modelling strategy was selected to maximize the ability to identify potentially relevant associations while minimizing the risk of overfitting associated with the small sample size.

A similar modelling approach was applied for the analysis of CRP levels using Tobit regression models. In these analyses, the CCV included baseline CRP level, age, and their interaction terms. Tobit regression models were also explored for APACHE II score, PCT and leukocyte level, whereas logistic regression models were used for the analysis of survival status (survival vs. death).

Variables showing potential association in univariable analyses were considered for inclusion in exploratory multivariable models, while keeping the number of covariates limited relative to the number of observations and events. Results were expressed as odds ratios (ORs) or hazard ratios (HRs), with 95% confidence intervals (CIs).

Overall survival was analyzed from the initiation of cefiderocol therapy using Kaplan–Meier curves and exploratory Cox proportional hazards models. Among the 25 included cases, 13 events were recorded. Given the limited number of events, each predictor variable was evaluated in a separate Cox regression model together with the core control variables. The proportional hazards assumption was assessed using Schoenfeld residuals.

A two-sided *p*-value < 0.05 was considered statistically significant, while *p*-values between 0.05 and 0.10 were considered indicative of a statistical trend. All descriptive analyses were performed using IBM SPSS Statistics version 27 (IBM Corp., Armonk, NY, USA), while predictive and survival analyses were conducted using Stata version 18 (StataCorp LLC, College Station, TX, USA).

## 5. Conclusions

In this real-world ICU case series, cefiderocol was associated with microbiological eradication in 68% of patients, along with improvement in severity scores and inflammatory markers in patients with MDR Gram-negative infections. Given the retrospective, uncontrolled design and limited sample size, causality cannot be established, and these findings are hypothesis-generating rather than confirmatory. They are nonetheless supportive of a potential role for cefiderocol as a salvage option in critically ill patients with MDR Gram-negative infections, pending validation in larger, prospective, controlled studies. Validation in larger, prospective, multicenter studies remains necessary before definitive conclusions can be drawn.

Clinical outcomes were primarily determined by baseline disease severity rather than pathogen characteristics alone, supporting the importance of early diagnosis and timely initiation of targeted antimicrobial therapy. Although limited by its retrospective single-center design and small sample size, this case series adds to the growing body of real-world evidence on cefiderocol use in complex ICU populations with severe MDR Gram-negative infections.

## Figures and Tables

**Table 1:** Demographic and clinical characteristics of patients treated with cefiderocol.

Characteristics	*N* = 25
Demographic, ICU and treatment characteristics	
Male	14 (56%)
Age, years, median (IQR)	53 (30–60)
Length of hospital stay, days, median (IQR)	46 (22–71)
Time from admission to infection confirmation, days, median (IQR)	21 (8–34)
Cefiderocol treatment duration, days, median (IQR)	8(7–14)
Monotherapy, n (%)	9 (34%)
Combined antibiotic therapy *, n (%)	16 (64%)
ARDS, n (%)	7 (28%)
Mechanical ventilation, median (IQR)	17 (5–41)
Surgical intervention, n (%)	14 (56%)
Primary diagnoses at hospital admission **, n (%)	
Gastrointestinal conditions	9 (36%)
Respiratory conditions	9 (36%)
Trauma/polytrauma	5 (20%)
Sepsis/septic shock	4 (16%)
Musculoskeletal issues	2 (8%)
Neurological conditions and other/hemorrhagic conditions	1 (4%)
Vasopressor treatments, n (%)	
Noradrenaline	18 (72%)
Vasopressin	12 (48%)
Dobutamine	1 (4%)
Type of infection, n (%)	
Ventilator associated pneumonia (VAP)	10 (40%)
Complicated intra-abdominal infections (cIAI)	4 (16%)
Soft tissues and skin structures infections (SSI)	2 (8%)
Urinary tract infections (UTI)	1 (4%)
VAP + cIAI	4 (16%)
VAP + UTI	4 (16%)
Severity scores, median (IQR)	
APACHE II	15 (12–19)
SOFA	3 (3–5)
Laboratory parameters	
WBC, 10^9^/L, median (IQR)	10.1 (9–19)
C-reactive protein, mg/L, median (IQR)	228 (200–272)
Procalcitonin, ng/mL, n (%)	
<0.5	1 (4%)
0.5–2	8 (32%)
2–10	8 (32%)
5–10	2 (8%)
>10	6 (24%)

Abbreviations: APACHE II, Acute Physiology and Chronic Health Evaluation II; ARDS, Acute Respiratory Distress Syndrome; IQR, interquartile; SOFA, Sequential Organ Failure Assessment; WBC, White Blood Cells. Notes: * Combined antibiotic therapy included colistin, linezolid, metronidazole, vancomycin, azithromycin, trimethoprim–sulfamethoxazole, eravacycline, and tigecycline; ** Some patients presented with two or three diagnoses. Laboratory parameters (WBC, CRP, PCT) reported reflect values at cefiderocol initiation.

**Table 2:** Isolate-level reconstruction of specimen type, target pathogen, cefiderocol susceptibility, and co-isolates, by patient (N = 25 patients, 52 isolates).

Patient No.	Specimen (Target Pathogen)	Target Pathogen	Cefiderocol Susceptibility	Co-Isolates/Notes
1	Wound secretion	*K. pneumoniae*	S	—
2	Bronchial aspirate	*K. pneumoniae* + *A. baumannii*	S (both)	*Proteus* spp.; *P. aeruginosa*
3	Urine culture	*K. pneumoniae*	S	Bronchial aspirate: Acinetobacter baumannii + *K. pneumoniae*—R, not treated
4	Bronchial aspirate	*K. pneumoniae*	S	—
5	Bronchial aspirate + blood culture	*Acinetobacter baumannii* + *Pseudomonas aeruginosa*	S (both)	*Enterobacter cloacae*—co-isolate, not treated
6	Wound secretion	*Klebsiella pneumoniae*	S	*A. baumannii*—co-isolate; *Enterococcus faecalis*—Gram-positive, not included in assessment
7	Urine culture	*K. pneumoniae*	S	—
8	Blood culture	*K. pneumoniae*	S	*E. coli*—co-isolate
9	Bronchial aspirate	K. pneumoniae	S (confirmed at inclusion)	*Enterococcus faecium—Enterococcus faecium*—Gram-positive, not included in assessment; *A. baumannii*—co-isolate
10	Bronchial aspirate	*K. pneumoniae*	S	*Enterobacter cloacae*; *P. aeruginosa*—co-isolates
11	Urine culture	*K. pneumoniae* + *A. baumannii*	S (both, confirmed at inclusion)	*P. aeruginosa*; *S. maltophilia*—co-isolates
12	Bronchial aspirate	*K. pneumoniae*	S	*P. aeruginosa*—co-isolate
13	Bronchial + wound + blood culture	*K. pneumoniae* (same organism, 3 sites)	S (all)	*A. baumannii*—co-isolate
14	Urine culture	*K. pneumoniae*	S (confirmed at inclusion)	*Cutibacterium acnes*—Gram-positive, not included in assessment
15	Bronchial aspirate + urine culture	*Acinetobacter baumannii*	S (both sites)	*K. pneumoniae*; *K. oxytoca*—co-isolates
16	Urine culture	*Acinetobacter baumannii*	S	*K. pneumoniae*—R, not treated
17	Bronchial aspirate	*K. pneumoniae*	S	*S. maltophilia*—co-isolate
18	Bronchial aspirate	*P. aeruginosa*	S (confirmed at inclusion)	*K. pneumoniae*; *Bacteroides fragilis*—co-isolates
19	Bronchial aspirate	*K. pneumoniae*	S	—
20	Bronchial aspirate	*Acinetobacter baumannii*	S	—
21	Bronchial aspirate	*K. pneumoniae*	S	—
22	Wound secretion (amputation stump)	*K. pneumoniae*	S	—
23	Wound secretion	*Acinetobacter baumannii*	S	*K. pneumoniae*; *Serratia marcescens*—co-isolates
24	Peritoneal fluid	*K. pneumoniae*	S	—
25	Urine culture	*K. pneumoniae*	S	—

S = susceptible; R = resistant; PDR = pandrug-resistant. Susceptibility not otherwise specified refers to cefiderocol. For Patients 9, 11, 14, and 18, cefiderocol susceptibility of the target pathogen was confirmed at treatment initiation per the inclusion criteria (Section 4.1); a complete original antibiogram for these isolates could not be retrieved from archived laboratory records, and co-isolate susceptibility is not reported for these cases. Target pathogen identity for these five patients was established from clinical documentation (admission and progress notes) rather than from the original laboratory report. Patients with the same target pathogen isolated from more than one specimen, or with more than one pathogen targeted from the same specimen, are noted accordingly.

**Table 3:** Resistance gene profiling.

Resistance Gene	*N* = 25 n (%)
NDM	13 (52%)
OXA-48	12 (48%)
CTX-M	10 (40%)
TEM	7 (28%)
SHV	6 (24%)
OXA-23	5 (20%)
VIM	5 (20%)
OXA-24	3 (12%)
AACA4	3 (12%)
SUL 1	3 (12%)
MEC-A	2 (8%)
QNRB	1 (4%)
QNRS	1 (4%)
KPC	1 (4%)
TETA	1 (4%)
Resistance mechanism categories	
MBL	1 (4%)
CLASS AMBER B + D	1 (4%)

Note: N = 25 refers to the number of patients. Individual patients may harbour more than one resistance gene and more than one pathogen; therefore, percentages represent the proportion of patients in whom each resistance gene was detected and do not sum to 100%. Abbreviations: AACA4 = aminoglycoside resistance; AMB B + D = Ambler beta-lactamase classification; CTX-M = extended-spectrum beta-lactamase; KPC = Klebsiella pneumoniae carbapenemase; MBL = metallo-beta-lactamase; MEC-A = methicillin resistance; NDM = New Delhi metallo-beta-lactamase; OXA-23/OXA-24/OXA-48 = oxacillinases; QNRB/QNRS = quinolone resistance genes; SHV = beta-lactamase gene (sulfhydryl variable); SUL1 = sulfonamide resistance gene; TEM = beta-lactamase gene; TETA = tetracycline resistance gene; VIM = metallo-beta-lactamase. Note: some patients may have two or more resistance genes. MBL and Ambler class B + D denote broader resistance mechanism/classification categories reported by the multiplex PCR assay when a specific gene target was not further resolved, rather than individual resistance genes, and are presented separately for clarity. Resistance genes reported in this table correspond to the designated target pathogen (see Table 2) in each patient, correlated with the corresponding clinical culture and antibiogram.

**Table 4:** Clinical and laboratory parameters at baseline and at the end of cefiderocol treatment.

Parameter	Initial (Initiation)	Final (End of Therapy)
APACHE II score, median (IQR)	15 (12–19)	10 (5–18)
SOFA score, median (IQR)	3 (3–5)	3 (0–5)
WBC, (×10^9^/L), median (IQR)	10 (9–19)	13 (8–17)
CRP, mg/L, median (IQR)	228 (200–272)	113 (75–246)
PCT, ng/mL, n (%)		
<0.5	1 (4%)	9 (36%)
0.5–2	8 (32%)	7 (28%)
2–10	8 (32%)	3 (12%)
5–10	2 (8%)	0 (0%)
>10	6 (24%)	6 (24%)

Abbreviations: APACHE II, Acute Physiology and Chronic Health Evaluation II; CRP, C-reactive protein; IQR, interquartile; PCT, procalcitonin; SOFA, Sequential Organ Failure Assessment; WBC, White Blood Cells.

## Data Availability

The original contributions presented in this study are included in the article. Further inquiries can be directed to the corresponding authors.

## Supplementary Materials

The following supporting information can be downloaded at https://www.mdpi.com/article/10.3390/pathogens15080786/s1: Table S1. Reconstruction of specimen type, target pathogen, cefiderocol susceptibility, and co-isolates at the patient level. Figure S1: Predicted probability of treatment effectiveness across age according to infection diagnosis ((a) respiratory, (b) gastrointestinal/intra-abdominal), compared with other diagnoses; shaded areas represent 95% confidence intervals; Figure S2: Predicted probability of treatment effectiveness across age according to screening culture results (a) negative, (b) ESBL+, (c) CRE+, (d) MRSA+, compared with other isolates without these resistance mechanisms; shaded areas represent 95% confidence intervals; Figure S3: Predicted final CRP level according to CRP level at cefiderocol initiation, stratified by admission diagnosis (septic shock/multiple sepsis), compared with other diagnoses; shaded areas represent 95% confidence intervals; Figure S4: Predicted probability of treatment efficacy according to clinical severity scores and inflammatory markers, stratified by gender (men vs. women): (a) APACHE II score at admission; (b) SOFA score at admission; (c) CRP level at cefiderocol initiation; (d) PCT level at cefiderocol initiation; shaded areas represent 95% confidence intervals; Figure S5: Predicted final SOFA scores according to SOFA at admission and baseline inflammatory markers, CRP (a) and PCT (b). Color gradients represent predicted final SOFA values, ranging from lower (dark) to higher (warm) scores. For example, a patient with high SOFA score and low CRP or PCT level at admission may achieve a low SOFA score at the end of cefiderocol treatment; Figure S6: Predicted final CRP levels showing the interaction between baseline CRP and age. Color gradients represent predicted CRP values (mg/L), ranging from lower (light) to higher (dark) levels. Higher baseline CRP values were associated with higher predicted final CRP levels, with age modifying this relationship. For example, elderly patients (>65 years) with high baseline CRP levels (>550 mg/L) were predicted to have lower final CRP levels (≤100 mg/L) compared with younger patients; Figure S7: Predicted probability plot of cure (vs. death) illustrating the interaction between age and body weight. Color gradients represent predicted probability of cure, ranging from high (light) to low (dark) probability.

## Abbreviations

AACA4	Aminoglycoside resistance
AMB B + D	Ambler beta-lactamase classification
AMR	Antimicrobial resistance
APACHE II	Acute Physiology and Chronic Health Evaluation II
ARDS	Acute respiratory distress syndrome
CI	Confidence interval
CR	Carbapenem-resistant
CR-AB	Carbapenem-resistant *Acinetobacter baumannii*
CR-E	Carbapenem-resistant Enterobacterales
CR-GNB	Carbapenem-resistant Gram-negative bacteria
CR-Kp	Carbapenem-resistant *Klebsiella pneumoniae*
CR-PA	Carbapenem-resistant *Pseudomonas aeruginosa*
CRP	C-reactive protein
CTX-M	Extended-spectrum beta-lactamase
ESBLs	Extended-spectrum β-lactamases
ESCMID	European Society of Clinical Microbiology and Infectious Diseases
EU/EEA	European Union/European Economic Area
GN	Gram-negative
HR	Hazard ratio
ICU	Intensive care unit
IDSA	Infectious Diseases Society of America
IQR	Interquartile range
KPC	*Klebsiella pneumoniae* carbapenemase
M/F	Male/Female
MBL	Metallo-beta-lactamase
MDR	Multidrug-resistant
MEC-A	Methicillin resistance gene (mecA)
MRSA	Methicillin-resistant *Staphylococcus aureus*
NDM	New Delhi metallo-beta-lactamase
OR	Odds ratio
OXA-23/OXA-24/OXA-48	Oxacillinases
PCT	Procalcitonin
PCR	Polymerase chain reaction
PDR	Pandrug-resistant
QNRB/QNRS	Quinolone resistance genes
SD	Standard deviation
SHV	Beta-lactamase gene (sulfhydryl variable)
SOFA	Sequential Organ Failure Assessment
SUL1	Sulfonamide resistance gene
TEM	Beta-lactamase gene
TETA	Tetracycline resistance gene
VIM	Metallo-beta-lactamase
VRE	Vancomycin-resistant Enterococci
WBC	White blood cells
WHO	World Health Organization
XDR	Extensively drug-resistant
