# Supplementary material for: Real-World Use of Cefiderocol for Multidrug-Resistant Gram-Negative Infections in Critically Ill ICU Patients: A Single-Center Case Series
Source: Pathogens. 2026 Jul 24;15(8):786. doi: 10.3390/pathogens15080786 (PMC13516722; doi:10.3390/pathogens15080786)
Supplement: Supplementary file 1 [file pathogens-15-00786-s001.zip › pathogens-4359724-supplementary.pdf]

Supplementary Table S1. Reconstruction of specimen type, target pathogen, cefiderocol susceptibility, and co-isolates at the patient level.

Patient No.	Specimen (target pathogen)	Target pathogen	Cefiderocol susceptibility	Co-isolates / notes
1	Wound secretion	*K. pneumoniae*	S	—
2	Bronchial aspirate	*K. pneumoniae* + *Providencia stuartii*	S (both)	—
3	Urine culture	*K. pneumoniae*	S	Bronchial aspirate: *Acinetobacter baumannii* + *K. pneumoniae* — R, not treated
4	Not reconstructable	Not reconstructable	S (confirmed at inclusion)	Original report not retrievable
5	Bronchial aspirate + blood culture	*Acinetobacter baumannii* + *Pseudomonas aeruginosa* (both targeted)	S (both)	*Enterobacter cloacae* — co-isolate, not treated
6	Wound secretion	*Klebsiella pneumoniae*	S	*Acinetobacter baumannii* — co-isolate
7	Urine culture	*K. pneumoniae*	S	—
8	Blood culture	*K. pneumoniae*	S	—
9	Not reconstructable	Not reconstructable	S (confirmed at inclusion)	Original report not retrievable
10	Bronchial aspirate	*K. pneumoniae* + *Stenotrophomonas maltophilia*	S (both)	Peritoneal fluid: *K. pneumoniae* (R) + *Corynebacterium* sp. — separate site, not treated
11	Not reconstructable	Not reconstructable	S (confirmed at inclusion)	Original report not retrievable
12	Bronchial aspirate	*K. pneumoniae*	S	—
13	Bronchial aspirate + wound + blood culture	*K. pneumoniae* (same organism, 3 sites)	S (all)	—
14	Not reconstructable	Not reconstructable	S (confirmed at inclusion)	Original report not retrievable
15	Bronchial aspirate + urine culture	*Acinetobacter baumannii*	S (both sites)	—
16	Urine culture	*Acinetobacter baumannii*	S	*K. pneumoniae* — R, not treated
17	Bronchial aspirate	*K. pneumoniae*	S	—
18	Not reconstructable	Not reconstructable	S (confirmed at inclusion)	Original report not retrievable
19	Bronchial aspirate	*K. pneumoniae*	S	*Acinetobacter baumannii* — R, not treated
20	Bronchial aspirate	*Acinetobacter baumannii*	S	—
21	Bronchial	*K. pneumoniae*	S	—

	aspirate			
22	Wound secretion (amputation stump)	*K. pneumoniae*	S	—
23	Wound secretion	*K. pneumoniae*	S	Same specimen: *Acinetobacter baumannii* + *Pseudomonas aeruginosa* (S); catheter tip: *K. pneumoniae* (R) — colonization, not treated
24	Peritoneal fluid	*K. pneumoniae*	S	—
25	Urine culture	*K. pneumoniae*	S	—

S = susceptible; R = resistant. For patients 4, 9, 11, 14, and 18, cefiderocol susceptibility of the causative pathogen was confirmed at treatment initiation (per inclusion criteria, Section 4.1), but the original specimen-level microbiology reports could not be retrieved from archived records for this retrospective reconstruction; specimen type and target pathogen identity are therefore not reported for these cases.

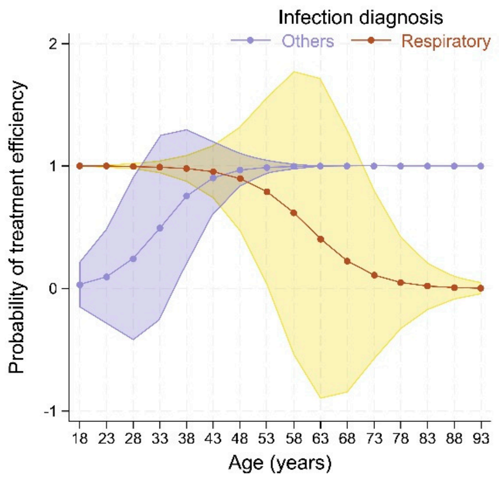

a)

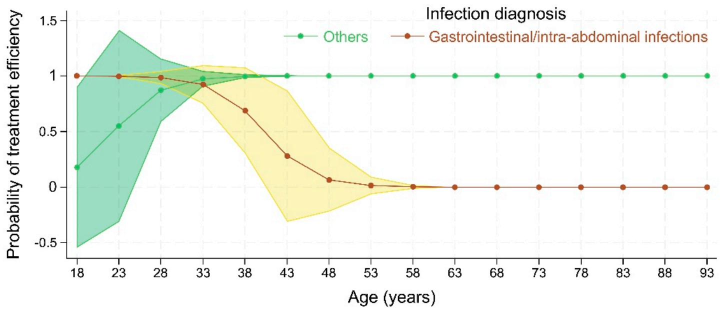

b)

Suppl. Figure S1: Predicted probability of treatment effectiveness across age according to infection diagnosis ((a) respiratory, (b) gastrointestinal/intra-abdominal), compared with other diagnoses; shaded areas represent 95% confidence intervals.

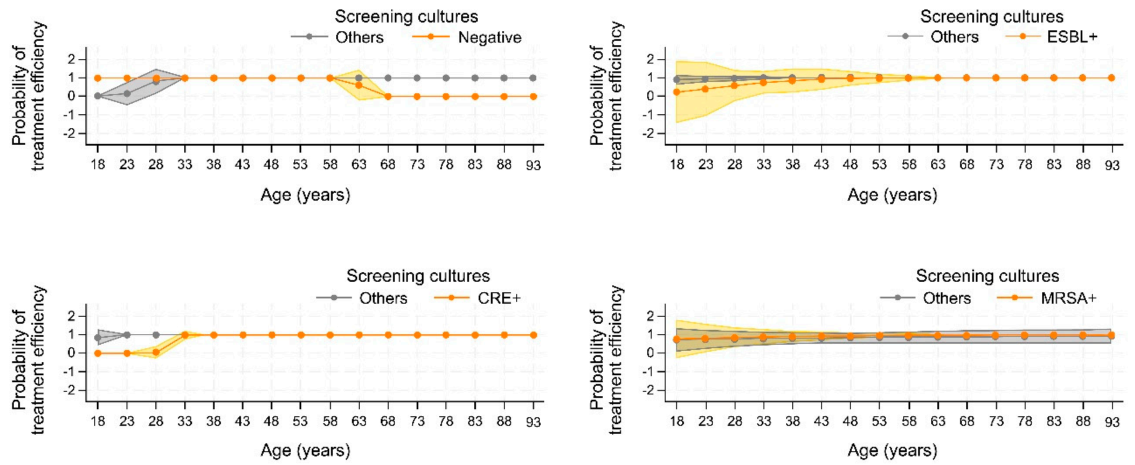

Suppl. Figure S2: Predicted probability of treatment effectiveness across age according to screening culture results (a) negative, b) ESBL+, c) CRE+, d) MRSA+), compared with other isolates without these resistance mechanisms; shaded areas represent 95% confidence intervals.

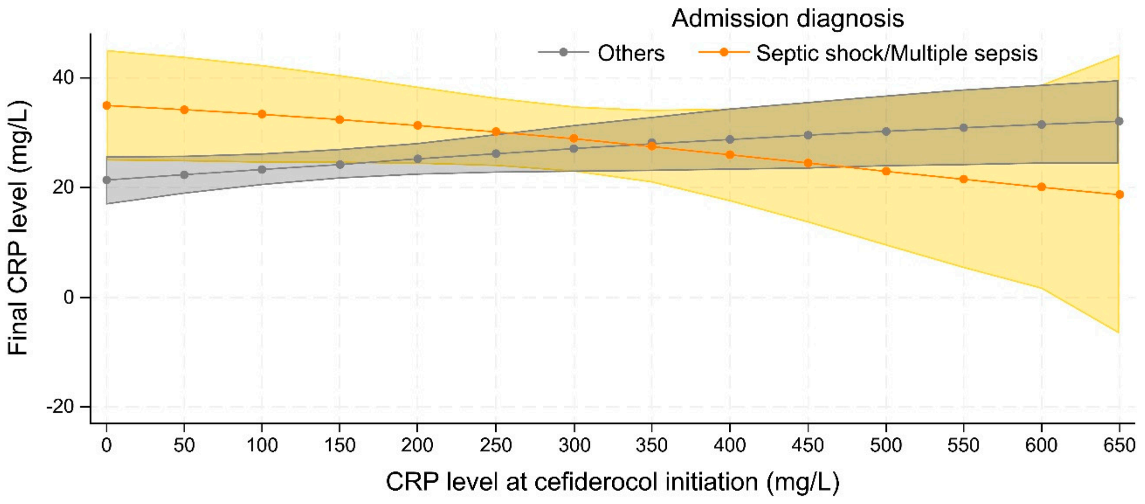

Suppl. Figure S3: Predicted final CRP level according to CRP level at cefiderocol initiation, stratified by admission diagnosis (septic shock/multiple sepsis), compared with other diagnoses; shaded areas represent 95% confidence intervals.

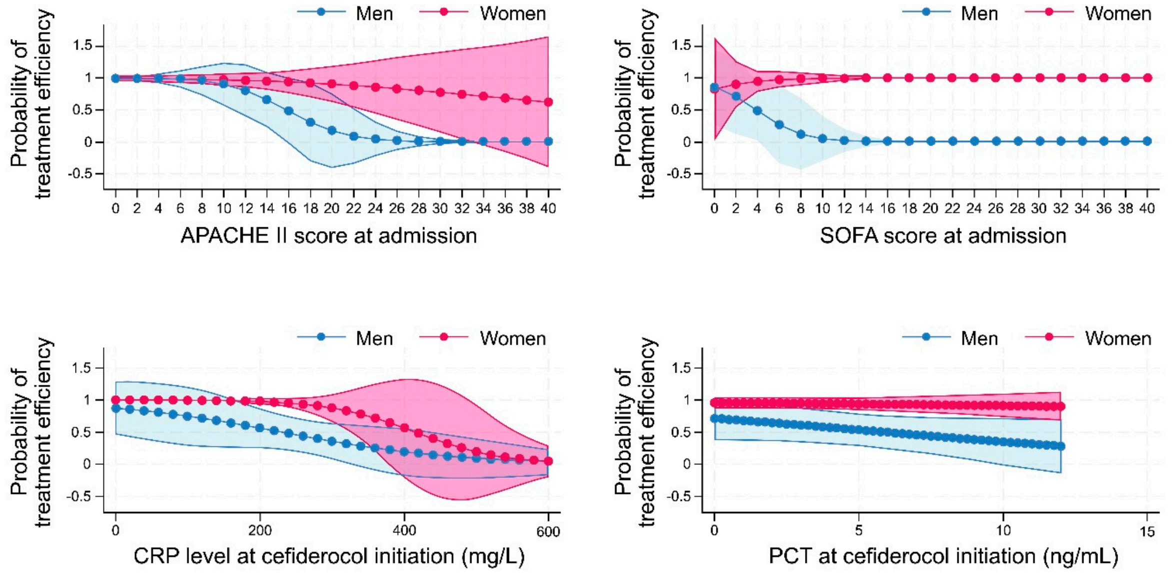

Suppl. Figure S4: Predicted probability of treatment efficacy according to clinical severity scores and inflammatory markers, stratified by gender (men vs. women): (a) APACHE II score at admission; (b) SOFA score at admission; (c) CRP level at cefiderocol initiation; (d) PCT level at cefiderocol initiation; shaded areas represent 95% confidence intervals.

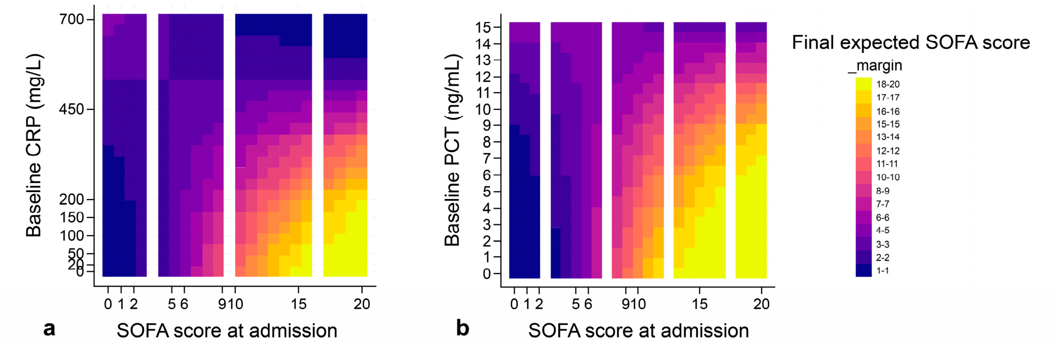

Suppl. Figure S5. Predicted final SOFA scores according to SOFA at admission and baseline inflammatory markers, CRP (a) and PCT (b). Color gradients represent predicted final SOFA values, ranging from lower (dark) to higher (warm) scores. For example, a patient with high SOFA score and low CRP or PCT level at admission may achieve a low SOFA score at the end of cefiderocol treatment.

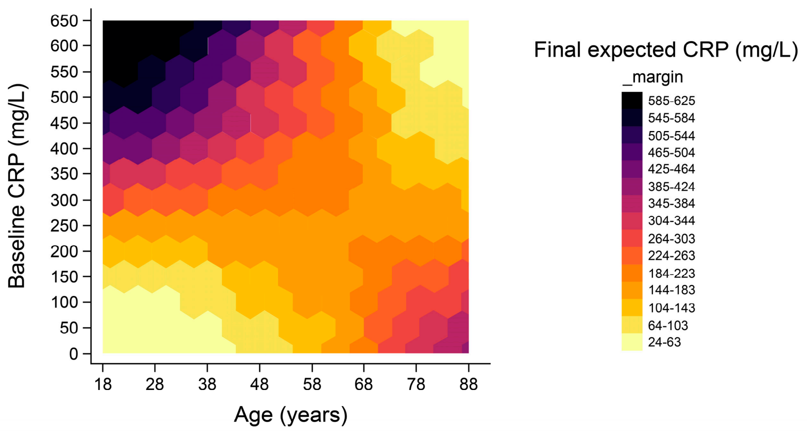

Suppl. Figure S6. Predicted final CRP levels showing the interaction between baseline CRP and age. Color gradients represent predicted CRP values (mg/L), ranging from lower (light) to higher (dark) levels. Higher baseline CRP values were associated with higher predicted final CRP levels, with age modifying this relationship. For example, elderly patients (>65 years) with high baseline CRP levels (>550 mg/L) were predicted to have lower final CRP levels (≤100 mg/L) compared with younger patients.

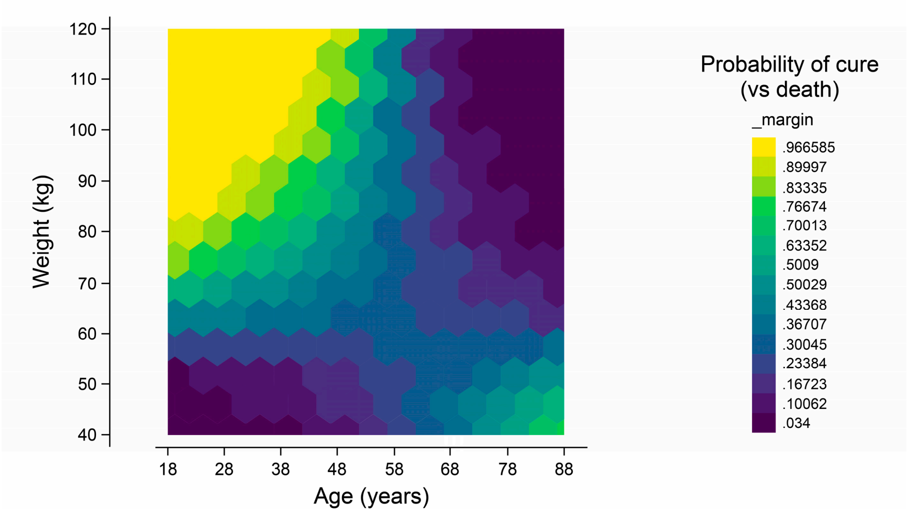

Suppl. Figure S7. Predicted probability plot of cure (vs. death) illustrating the interaction between age and body weight. Color gradients represent predicted probability of cure, ranging from high (light) to low (dark) probability.
